# Caregivers’ Perspectives on Their Self-Care Practices While Caring for Older Adults With Chronic Illness

**DOI:** 10.1093/geroni/igaf122.799

**Published:** 2025-12-31

**Authors:** Soojung Ahn, Kemberlee Bonnet, Ashley Sellers, Christopher Lee, Mulubrhan Mogos

**Affiliations:** Boston College William F. Connell School of Nursing, Chestnut Hill, Massachusetts, United States; Vanderbilt University, Nashville, Tennessee, United States; Vanderbilt University, Nashville, Tennessee, United States; Boston College William F. Connell School of Nursing, Chestnut Hill, Massachusetts, United States; Vanderbilt University, Nashville, Tennessee, United States

## Abstract

The demands of family caregiving can significantly impede self-care practices, often resulting in self-neglect, health decline, diminished quality of life, and vulnerability to chronic illness. Despite these impacts, few studies have investigated the underlying factors contributing to caregivers’ diminished self-care practices. This study aimed to explore caregivers’ perspectives on barriers and facilitators to their self-care practices. We conducted four focus groups with caregivers primarily responsible for providing in-home care to older adults with chronic illnesses. Sessions were audio-recorded, transcribed, and analyzed using a hierarchical coding system refined by experienced qualitative researchers through an iterative inductive-deductive approach. Five major themes emerged: 1) varied definitions and perceptions of self-care practices among caregivers; 2) psychosocial barriers to self-care practices, including emotional strain, cognitive demands of caregiving, maladaptive or avoidant coping, and loss of sense of control; 3) contextual barriers, including caregiving roles, the health status of the care recipient, family dynamics, and resource availability; 4) social support and knowledge of resources as facilitators to better self-care; and 5) problem-based coping strategies developed by caregivers, such as systems for organization, to regain sense of control and alleviate stress. These findings underscore the importance of understanding caregivers’ perceptions and the multi-level factors influencing their self-care practices. Future research should focus on identifying and implementing strategies to mitigate these barriers, ultimately fostering better self-care among caregivers to improve their quality of life and health outcomes.

